# Mechanical Properties of Weld Lines in Injection-Molded Carbon Fiber-Reinforced Nylon (PA-CF) Composites

**DOI:** 10.3390/polym15112476

**Published:** 2023-05-27

**Authors:** Xianpeng Wang, Zuguo Bao, Donglin Gao, Shiyao Huang, Li Huang, Qiuren Chen, Hailong Zhao, Weijian Han, Yahong Xu

**Affiliations:** 1 College of Materials Science and Engineering, Nanjing Tech University, Nanjing 210009, China; 2 Yangtze Delta Region Institute of Advanced Materials, Suzhou 215133, China

**Keywords:** injection molding, weld line, fiber orientation, PA-CF composites

## Abstract

Weld lines are a common defect generated in injection molding, which apparently affects the performance of final products, but the available reports on carbon fiber-reinforced thermoplastics are still rather few. In this study, the effects of injection temperature, injection pressure, and fiber content on the mechanical properties of weld lines were studied for carbon fiber-reinforced nylon (PA-CF) composites. The weld line coefficient was also calculated by comparing specimens with and without weld lines. The tensile and flexural properties of PA-CF composites significantly increased with the rise of fiber content for specimens without weld lines, while injection temperature and pressure demonstrated slight influences on mechanical properties. However, the existence of weld lines had negative influences on the mechanical properties of PA-CF composites due to poor fiber orientation in weld line regions. The weld line coefficient of PA-CF composites decreased as fiber content increased, indicating that the damage of weld lines to mechanical properties increased. The microstructure analysis showed that there were a large number of fibers distributed vertically to flow direction in weld lines regions, which could not play a reinforcing role. In addition, increasing injection temperature and pressure facilitated fiber orientation, which improved the mechanical properties of composites with low fiber content, while weakening composites with high fiber content instead. This article provides practical information for product design containing weld lines, which helps to optimize the forming process and formula design of PA-CF composites with weld lines.

## 1. Introduction

Weld lines have been one of the common defects puzzling the injection molding industry. When two or more strands of molten plastics meet in the mold, a weld line will be produced at the junction [1,2]. Weld lines not only affect the appearance and quality of products, but also cause negative influences on the mechanical properties [3,4,5]. Such influences were especially obvious for fiber-reinforced thermoplastics, which have been widely used in the automobile and home appliance industries in recent years [6]. In the weld line regions of fiber-reinforced composites, most fibers were parallel to the interface where the melt intersected, and failed to play a reinforcing role [7,8,9]. Thus, the weld lines are usually known as weak regions in the products. However, for the consideration of production efficiency and economic benefits, multiple gate designs were often employed in injection molding, inevitably producing weld lines on the products. Therefore, it is necessary to investigate the properties of weld lines and their contributory factors.

It has been reported that the mechanical properties of weld line regions would be enhanced with the increase in injection temperature and mold temperature [10,11,12]. In addition, with the increase in the injection pressure, the mechanical properties also showed an increasing trend [13,14]. However, the tensile strength and fracture strain would decrease significantly with the increase in the fiber content in weld line regions [15,16,17]. Andrea Scantamburlo prepared 35wt% glass fiber reinforced polypropylene tensile specimens with weld lines using alternate dynamic fillers and rapid heat cycle injection molding (RHCM). Compared with traditional injection molded weld lines, the RHCM reduced the skin layer thickness of the specimens, and increased the strength and stiffness of the specimens by 201% and 46%, respectively [18]. Babur Ozcelik analyzed the effects of injection temperature and injection pressure on the mechanical properties of polypropylene (PP) specimens with and without weld lines. When the injection temperature and injection pressure increased, the tensile strength of the specimens with and without weld lines did not change significantly, but the impact resistance decreased significantly [19]. Azieatul Azrin Dzulkipli studied the effect of fiber addition on the formation of weld lines. Glass fiber-reinforced polypropylene had a shorter weld line length and a larger meeting angle than pure polypropylene [12]. Gyung-Hwan Oh investigated the effect of fiber content on the tensile strength of specimens with and without weld lines in glass fiber-reinforced modified polyphenylene oxide (MPPO) composites. For tensile specimens with glass fiber weight percentages of 0%, 10%, 20%, and 30%, the proportion of strength reductions due to the existence of weld lines were 12%, 34%, 52%, and 56%, respectively [20]. L.M. Martulli investigated the effect of weld lines on the mechanical properties of carbon fiber tow-reinforced vinyl ester sheet molding compound (CF-SMC) with a fiber volume fraction of 42%, and found that the tensile strength of the sample with weld lines was 48% to 88% of that without weld lines [21]. R. Selden focused on the influence of injection temperature, mold temperature, packing pressure, and injection speed on the flexural strength of five types of thermoplastics (ABS, PPO, PA6, PPS, and PP containing 40% talc) with/without weld lines, and calculated the weld line coefficient, which varied from 0.25 to 0.98 in the tested materials [22].

Currently, research on weld lines is mostly focused on pure plastics and glass fiber-reinforced plastics, and there are fewer reports on the weld lines of PA-CF composites. Compared with glass fiber, carbon fiber reinforced composites possess more excellent properties and broader potential for structural applications [23]. As the weld lines are typically weak regions in injection molding parts, the investigation of weld line properties is very critical for the design and process optimization when using this material to prepare products with weld lines. The purpose of this work is to investigate the mechanical properties of weld lines in PA-CF composites, considering the influence of injection temperature, injection pressure, and fiber content. Weld line coefficients were also calculated. Scanning electron microscope (SEM) and metallographic microscope were used to observe the microstructure of weld line samples. The software Moldex3D was employed to simulate the injection molding process and analyze the fiber orientation in the weld line regions.

## 2. Materials and Methods

### 2.1. Materials

Carbon fiber reinforced nylon 66 pellets were purchased from SABIC (No. RE0069S, SABIC Innovative Plastics Co., Ltd., Thorndale, TX, USA) with a fiber content of 30wt% (hereafter “PA-30CF”), and the nominal material properties are listed in Table 1. Due to the water absorption of nylon matrix, the raw material and specimens were dried for 4 h at 80 °C before injection molding and property testing [24,25].

### 2.2. Injection Molding

An injection molding machine, HAITIAN-MA900Ⅲ/280-A (Haitian Plastic Machinery Group Co., Ltd., Ningbo, China), was employed for the specimen preparation. The maximum clamping force was 90 tons, and it was equipped with a screw with a diameter of 32 mm and length–diameter ratio of 22.5. The setting of injection molding parameters is shown in Table 2, in which the influence of injection temperature and injection pressure on the mechanical properties of the specimens with/without weld lines were considered. The mold temperature, packing pressure, and injection speed were maintained constant with the value of 25 °C, 8 MPa, and 70 mm/s, respectively. In addition, the influence of fiber content was also considered with four loading levels, namely 0%, 10%, 20%, and 30% by weight. The specimens with lower fiber contents were prepared by mixing PA-30CF composites and pure PA proportionally in injection molding.

### 2.3. Specimens

**Figure 1:**
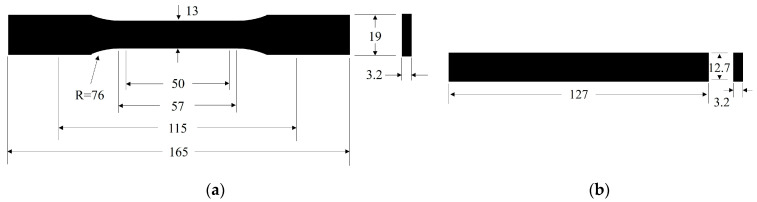
Specimens: (**a**) tensile testing specimen and (**b**) flexural testing specimen.

**Figure 2:**
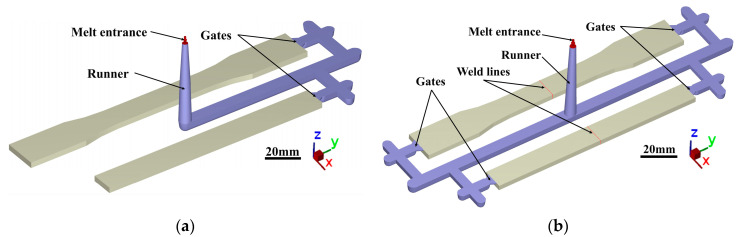
The injection runners of the specimens (**a**) without weld lines and (**b**) with weld lines.

The tensile specimen was designed according to ASTM D638 Type I, as shown in Figure 1a. The total length of tensile specimen was 165 mm, with the gauge length of 50 mm. The flexural specimen (Figure 1b) was designed according to ASTM D790, with a span width of 51.2 mm. The injection runners of the specimens are exhibited in Figure 2. For the specimen without weld lines, there was only a gate at one end of the cavity for the melt to flow in, as shown in Figure 2a. For the specimen with weld lines, both ends of the cavity were equipped with gates, and the two streams of melt converged in the middle area of the specimen and formed the weld line, as shown in Figure 2b.

### 2.4. Mechanical Properties Testing

Mechanical properties, including tensile and flexural properties, were tested in INSTRON 5982 (Instron Co., Ltd., Boston, MA, USA). The tensile testing was conducted according to ASTM-D638, with a loading speed of 5 mm/min. The flexural testing was conducted according to ASTM-D790, with a loading speed of 1.4 mm/min. Five duplicate specimens were tested for each material, and the mean values were provided.

The weld line coefficient $F_{kl}$ was used to describe the influence of weld lines on the mechanical properties of specimens, and the calculation formula is shown in (1) [12,26].

(1) $$F_{kl}=\frac{\sigma_{WL}}{\sigma_{NWL}}$$

where $\sigma_{WL}$ and $\sigma_{NWL}$ are mechanical properties with and without weld lines, respectively.

### 2.5. Microscopic Observation

**Figure 3:**
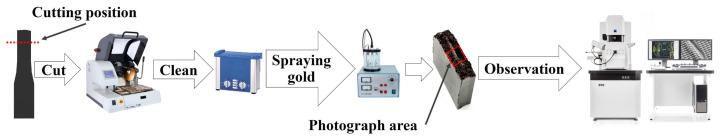
SEM observation process.

**Figure 4:**
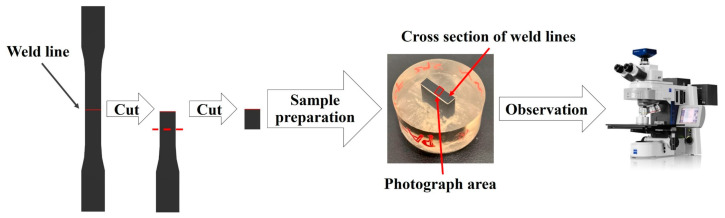
Metallographic microscope observation process.

Fracture morphology was observed with the aid of scanning electron microscope (SEM) for the specimen after failure. The operation process is shown in Figure 3. The fiber orientation and distribution in the weld line regions were observed with ZEISS-Axio Imager M2m (Carl Zeiss Co., Ltd., Oberkohen, Germany) microscope, and the operation process is shown in Figure 4.

### 2.6. DSC Measurement

The samples in the weld line regions were cut from the tensile specimens and analyzed by differential scanning calorimetry (DSC). The DSC measurement was conducted in TA-DSC25 (TA instrument Co., Ltd., New Castle, DE, USA) differential scanning calorimeter to investigate the crystallinity of nylon in samples. The samples were heated from 25 °C to 330 °C with a heating rate of 10 °C/min and kept for 5 min, and then cooled to 25 °C with a cooling rate of 5 °C/min.

### 2.7. Injection Molding Simulation

**Figure 5:**
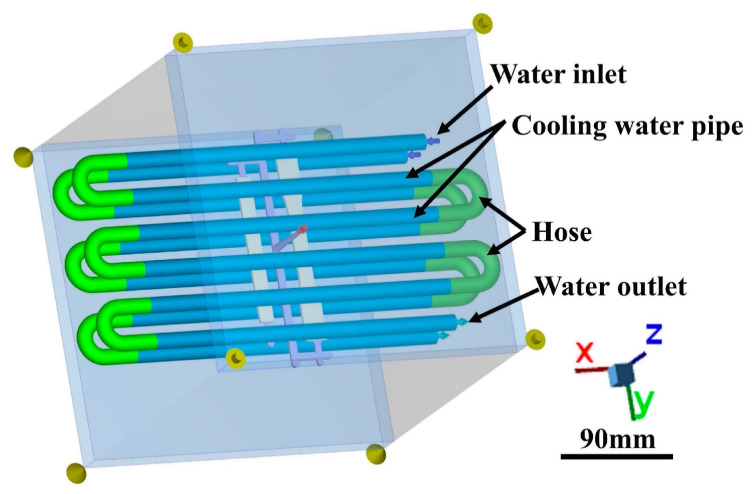
Moldex3D simulation model.

The mold flow analysis software Moldex3D Studio 2021 (CoreTech System Co., Ltd., Suzhou, China) was used to simulate the injection molding process, including filling, packing, cooling, and other stages, to analyze the fiber orientation tensor distribution, melt meeting angle, etc. The design of the cooling water path of the model is shown in Figure 5.

Melt flow is a non-isothermal process in injection molding. The melt is a typical non-Newtonian fluid, and its viscosity is affected by the temperature and shear rate changes during the filling process [27,28,29]. Thus, the above factors were taken into account, and Cross-WLF viscosity model was selected, as shown in Formulas (2)–(5) [30,31]. This model integrated the effects of temperature, pressure, and shear rate on the viscosity of melt, and could accurately predict the flow process accompanied by cooling effects. The values of material parameters are shown in Table 3.

(2) $$\eta =\frac{\eta_{0}}{1+{\left(\frac{\eta_{0}\dot{\gamma }}{\tau^{*}}\right)}^{1-n}}$$

where η and $\eta_{0}$ are viscosity and zero shear viscosity, respectively; $\dot{\gamma }$ is shear strain rate, $\tau^{*}$ represents the critical stress level at the transition to shear thinning, n is the power law index in the high shear rate regime.

(3) $$\eta_{0}=D_{1}exp\left[\frac{-A_{1}\left(T-T_{c}\right)}{A_{2}+\left(T-T_{c}\right)}\right]$$

where $D_{1}$ and $A_{1}$ are data-fitted coefficients, *T* is the melting point temperature of the material, $T_{c}$ is the reference temperature, and the glass transition temperature of the material is generally selected.

(4) $$T_{c}=D_{2}+D_{3}P$$

(5) $$A_{2}=\tilde{A_{2}}+D_{3}P$$

where $D_{2}$, $D_{3}$, and $\tilde{A_{2}}$ are data-fitted coefficients; P is the pressure.

## 3. Results and Discussion

### 3.1. Effect of Injection Temperature on Mechanical Properties

**Figure 6:**
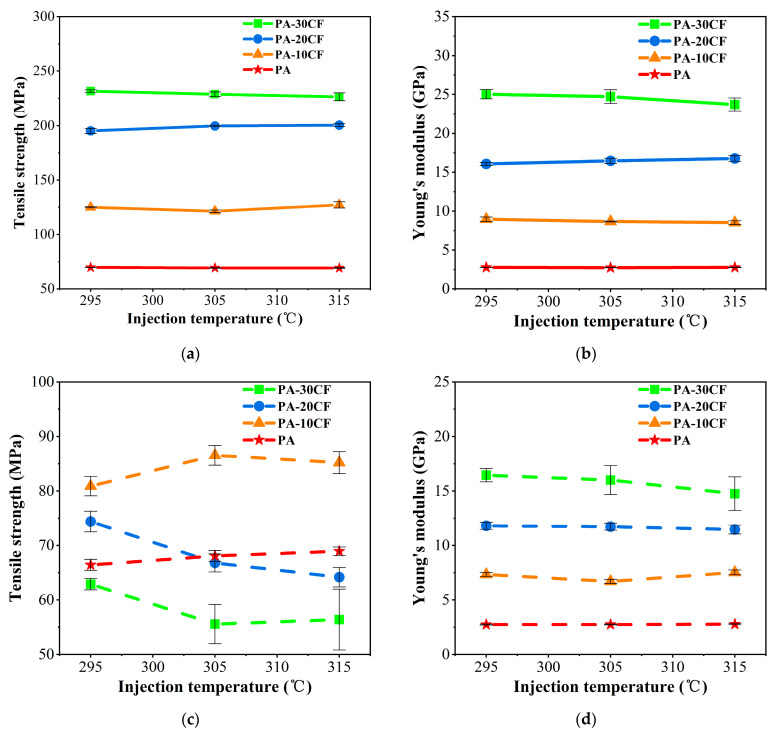
Tensile properties of PA-CF composites for different fiber contents and injection temperatures: (**a**) tensile strength, without weld lines; (**b**) Young’s modulus, without weld lines; (**c**) tensile strength, with weld lines; and (**d**) Young’s modulus, with weld lines.

Mechanical properties of PA-CF composites were measured for different fiber contents and injection temperatures. As shown in Figure 6a,b, the tensile strength and Young’s modulus of PA-CF composites without weld lines were significantly elevated with the increase in fiber content, indicating fibers played a reinforcing role for the nylon matrix. When carbon fiber reached 30wt%, the tensile strength and Young’s modulus of PA-CF composites were 2.32 and 8.01 times higher than that of pure nylon, respectively (injection molding at 295 °C). Overall, the injection temperature had little effect on the tensile strength and modulus of PA-CF composites without weld lines, which was similar to the results of many previous studies [32,33]. Carbon fibers are the main load-bearing component in PA-CF composites, and their orientation distribution determines the mechanical properties of PA-CF composite materials. Most fibers were oriented along the flow direction in the specimens without weld lines, and the influence of injection temperature on fiber orientation was limited. Therefore, changing the injection temperature did not significantly change the mechanical properties of PA-CF composites.

However, the existence of weld lines had negative influences on the tensile properties of PA-CF composites. As shown in Figure 6c,d, the specimens with weld lines had significantly lower tensile strength and Young’s modulus than those without weld lines. Meanwhile, it is worth noting that PA-20CF and PA-30CF composites had obviously lower tensile strength than PA-10CF, and PA-30CF was even lower than pure nylon, indicating that a large proportion of fibers did not play a reinforcing role, which was similar to the results of many previous studies [34,35]. Only fibers passing through the interface where the melts intersected could have a reinforcing effect, while higher fiber content hindered this process and reduced the entanglement between polymer chains. Additionally, the tensile strength of PA-20CF and PA-30CF composites with weld lines showed a downward trend when injection temperature increased (Figure 6c), while PA-10CF showed an upward trend. Compared with the tensile strength of the specimen at 295 °C, PA-20CF decreased by 10.2% and 13.7%, and PA-30CF decreased by 11.7% and 10.4%, while PA-10CF increased by 6.9% and 5.3% at 305 °C and 315 °C, respectively. The resin melt had better fluidity at higher injection temperatures, which was beneficial for fiber orientation [36]. The reinforcement effect was more obvious for those with low fiber content. A large proportion of fibers arranged parallel to the interface of the weld line for those with high fiber contents, which had greater adverse influences on the properties of composites and led to a decline in strength. In addition, samples with higher fiber content had a higher Young’s modulus, which was rarely affected by injection temperature.

**Figure 7:**
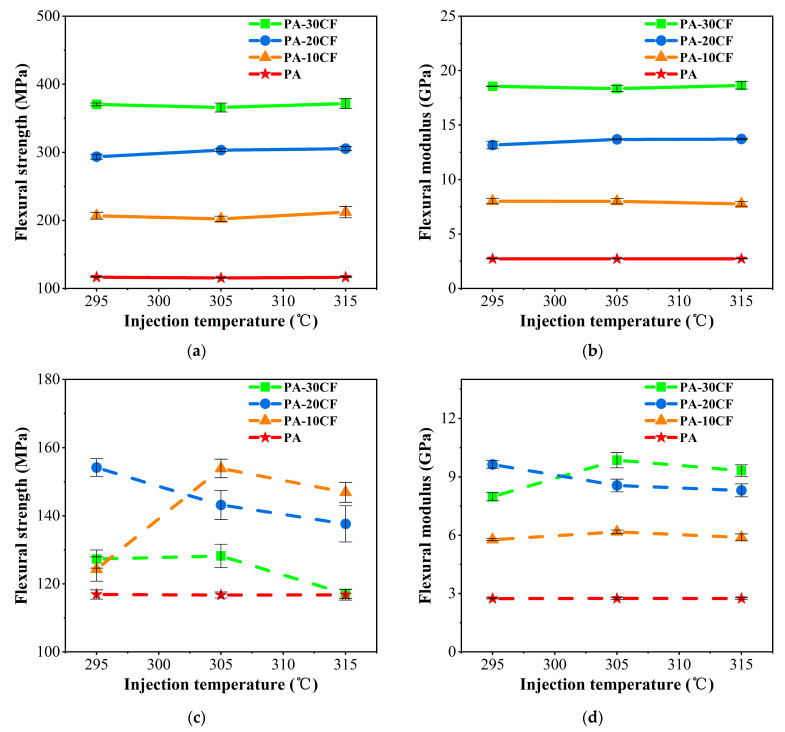
Flexural properties of PA-CF composites for different fiber contents and injection temperatures: (**a**) flexural strength, without weld lines; (**b**) flexural modulus, without weld lines; (**c**) flexural strength, with weld lines; and (**d**) flexural modulus, with weld lines.

Flexural properties of PA-CF composites were also measured for different fiber contents and injection temperatures. Similar to tensile properties, the flexural strength and flexural modulus of PA-CF composites without weld lines significantly increased with the rise of fiber content for specimens, as shown in Figure 7a,b. The flexural strength and flexural modulus of PA-CF composites increased by 3.18 and 6.81 times, respectively, compared to pure nylon (injection molding at 295 °C) when carbon fiber reached 30wt%. The injection temperature had a negligible influence on the flexural strength and modulus of PA-CF composites without weld lines as well.

Similar to tensile properties, flexural properties of PA-CF composites were also significantly influenced by the introduction of weld lines. As shown in Figure 7c,d, the specimens with weld lines had lower flexural strength and modulus than those without weld lines. The flexural strength and modulus of the samples containing carbon fibers were higher than those of pure nylon. As the injection molding temperature increased, the flexural strength of PA-CF composites was also affected, which was similar to the effect of temperature on the tensile strength of PA-CF composites. When injection temperature reached 305 °C or 315 °C, PA-10CF composites had higher flexural strength than that of PA-20CF and PA-30CF, indicating that the reinforcement effect of fibers was limited in samples with weld lines.

To further analyze the influences of injection temperature and fiber content on the mechanical properties of PA-CF composites, weld line coefficients were calculated according to Formula (1) $F_{kl}=\frac{\sigma_{WL}}{\sigma_{NWL}}$ and are listed in Table 4. The values of pure nylon were very close to 1, indicating that the weld line had a negligible influence on its mechanical properties. With the introduction of carbon fiber, the weld line coefficient was significantly affected, and their values apparently decreased with the rise of fiber content. It demonstrated that the weld line had a greater influence on the mechanical properties of PA-CF composite when fiber content increased. With the same carbon fiber content, the tensile strength was more severely affected by weld lines than flexural strength with lower weld line coefficients, while the flexural modulus was more sensitive to weld lines than Young’s modulus.

**Figure 8:**
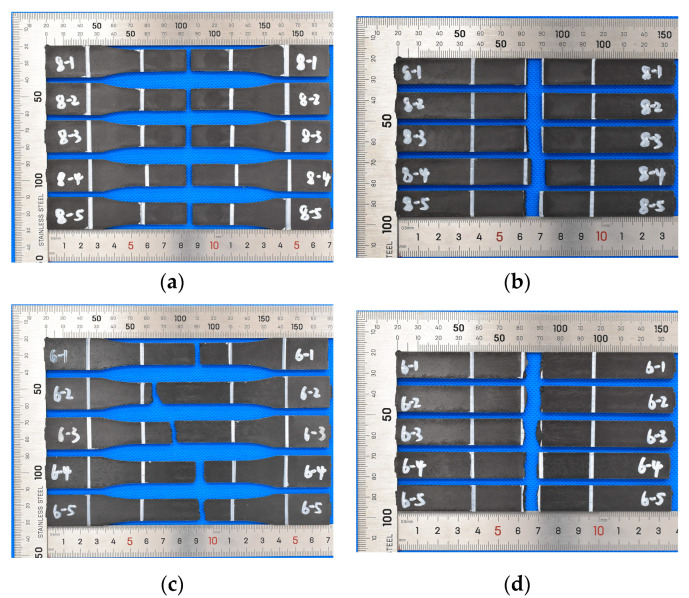
Fracture position of specimens with/without weld lines (injection molding at 295 °C): (**a**) tensile fracture, with weld lines; (**b**) flexural fracture, with weld lines; (**c**) tensile fracture, without weld lines; and (**d**) flexural fracture, without weld lines.

As the weld lines were the weak regions of specimens, their existence would affect the failure mode of the PA-CF composite. The fracture of specimens tended to occur close to the weld lines, as shown in Figure 8a,b. The fracture was relatively flat, and there were almost no small fragments produced by damage, indicating that weld lines were weak regions of material properties. The failure positions of specimens without weld lines are shown in Figure 8c,d. The fracture position was randomly located in the gauge length with a rough fractural surface and small fragments produced during the damage.

**Figure 9:**
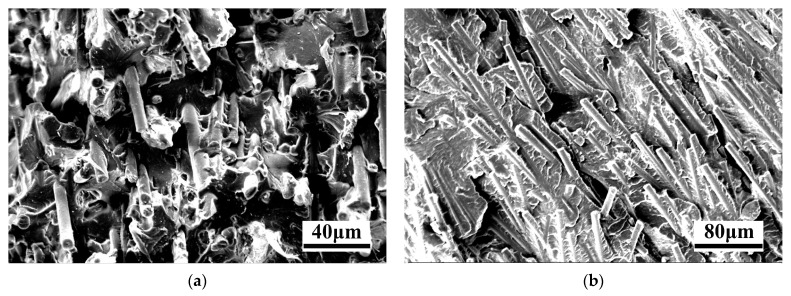
Fracture morphology of tensile specimens of PA-30CF composites (injection molding at 295 °C) (**a**) without weld lines and (**b**) with weld lines.

The fracture morphology of PA-30CF composites was observed with SEM and is illustrated in Figure 9, in which the flow direction was perpendicular to the observation surface. According to Figure 9a, the fibers of the sample without weld lines were oriented along the flow direction, and its failure modes included fiber pulling out, fiber breakage, and resin matrix tearing. As shown in Figure 9b, a large number of carbon fibers of the sample with weld lines were oriented perpendicular to the flow direction, causing the dominant failure modes in the weld line region to be the debonding of the matrix and the fibers, and the fracture of the resin. Most carbon fibers failed to play a reinforcing role, deteriorating the mechanical properties of PA-CF composites with high fiber content.

**Figure 10:**
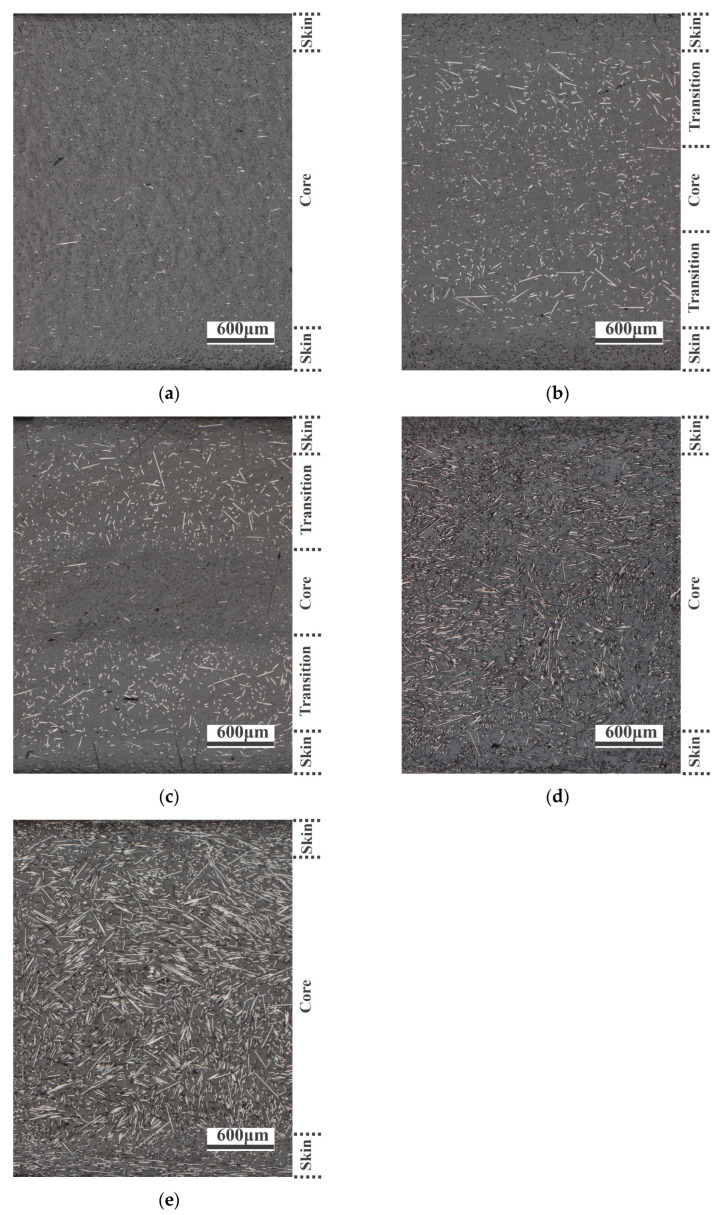
Metallographic micrograph (**a**) without weld lines, PA-10CF, 305 °C; (**b**) with weld lines, PA-10CF, 295 °C; (**c**) with weld lines, PA-10CF, 305 °C; (**d**) with weld lines, PA-30CF, 295 °C; and (**e**) with weld lines, PA-30CF, 305 °C.

The microscopic images of PA-CF composites are shown in Figure 10, and the flow direction was perpendicular to the observation surface as well. As shown in Figure 10a, most of the fibers were oriented along the flow direction for specimens without weld lines. This structure could fully exert the reinforcement effect of fibers, and the composites obtained good mechanical properties. As shown in Figure 10b,c, in the samples with weld lines, the region presented a skin–transition–core structure, wherein the fibers of the skin layer were mainly oriented along the flow direction, while in the transition layer, a large proportion of fibers were perpendicular to the flow direction, which had negative influences on the mechanical properties of materials. The degree of fiber orientation in the core layer was intermediate between the skin layer and the transition layer. It is worth noting that the fibers in the core layer tend to orient along the flow direction when the injection temperature increases, which is beneficial for improving mechanical properties. Meanwhile, the fiber orientation in weld line regions of PA-30CF composites was observed as well, as shown in Figure 10d,e. The transition and core layers were fused together when the fiber content was high. A large number of fibers were oriented perpendicular to the flow direction, and the reinforcement effect of fibers in this structure on the matrix was very weak. At the same time, this phenomenon became more obvious with the rise of injection temperature, which did greater damage to the mechanical properties of materials. The changes in the microstructure of the weld line regions validated the previous results of mechanical properties well.

**Figure 11:**
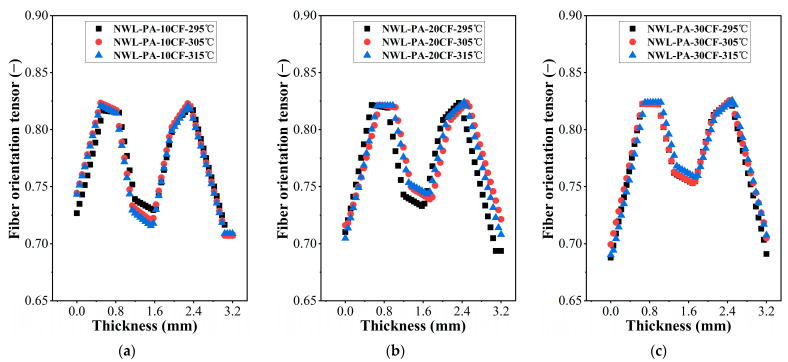

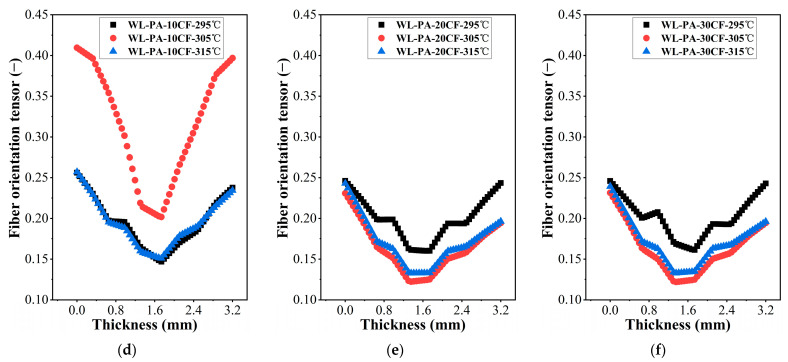
Fiber orientation tensors of specimens with/without weld lines for different injection temperatures: (**a**) without weld lines, PA-10CF; (**b**) without weld lines, PA-20CF; (**c**) without weld lines, PA-30CF; (**d**) with weld lines, PA-10CF; (**e**) with weld lines, PA-20CF; and (**f**) with weld lines, PA-30CF.

The fiber orientation tensor could describe the probability of fiber orientation in a specified principal direction. A value close to 1 indicates a high probability of fiber orientation in the specified principal direction, while a value close to 0 indicates a low probability. The distribution of fiber orientation tensor components in the length direction of specimens obtained by simulation is shown in Figure 11, and the tensors were approximately symmetrical along the thickness direction. The orientation tensors of samples without weld lines are shown in Figure 11a–c. During the transition from the skin layer to the core layer, the orientation tensors first increased and then decreased, which was similar to the results of many previous studies [37,38]. The skin layer contacted the cavity, and the lower temperature during the filling process led to rapid condensation of the melt, which was not conducive to fiber reorientation. However, the core layer had a higher temperature, resulting in better melt fluidity, and the fibers were subjected to complex shear effects, which were not conducive to the orientation of the fibers along the flow direction. As the fiber content increased, the interaction between fibers became more pronounced, and the tensors of the skin layer decreased as the fiber content increased, while the core layer exhibited the opposite behavior. The injection temperature hardly influenced the fiber orientation tensors.

The fiber orientation tensors in weld line regions are shown in Figure 11d–f, in which the skin layer had larger tensors than the core layer. When two strands of melts intersected, there existed a flow process from the core layer to the skin layer, which drove the fibers in the core layer to orient perpendicularly to the initial flow direction, resulting in a lower orientation tensor of the fibers in the core layer. PA-10CF composites had the largest orientation tensor at 305 °C, while PA-20CF and PA-30CF had the largest orientation tensor at 295 °C. A larger fiber orientation tensor means better reinforcement of fibers for the matrix, corresponding to better mechanical properties. The simulation results of the fiber orientation tensor validated the change in the mechanical properties of the specimens well.

**Figure 12:**
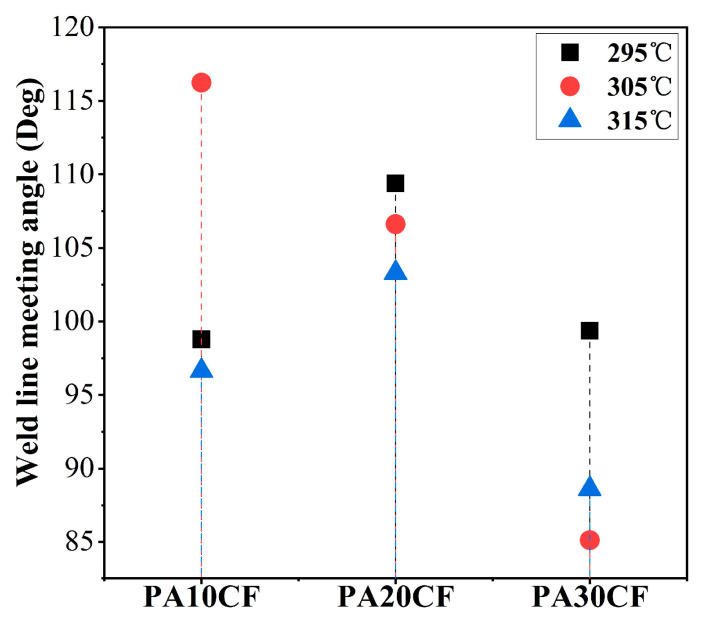
Weld line meeting angle for different fiber contents and temperatures.

It was reported that the weld line meeting angle was related to the fiber orientation and mechanical properties of specimens [2]. Generally, a larger meeting angle causes higher mechanical properties and a more inconspicuous appearance of the weld line. The simulation results of the weld line meeting angle of PA-CF composites are exhibited in Figure 12. PA-10CF composites achieved the largest meeting angle at 305 °C, and PA-20CF and PA-30CF achieved the largest meeting angle at 295 °C. Larger weld line meeting angles corresponded to better mechanical properties as well.

**Figure 13:**
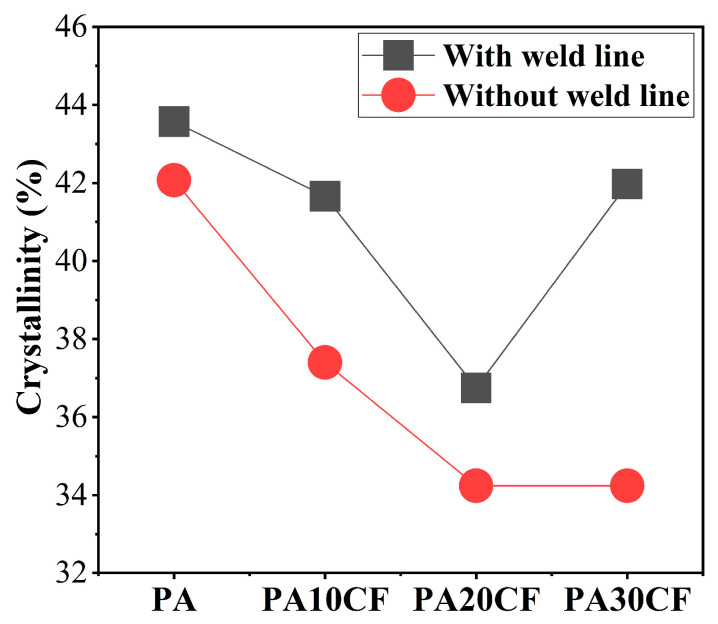
Crystallinity of specimens with/without weld lines for different fiber contents.

In addition to the fiber orientation, the crystallinity of nylon in the weld line regions was also investigated. The results obtained by DSC measurement are shown in Figure 13. Generally, the crystallinity of nylon in the samples with weld lines was higher than those without weld lines, indicating that the presence of weld lines promoted the crystallization of nylon. However, the mechanical properties of the samples with weld lines were still lower than those without weld lines. Combined with the previous microstructure analysis, it was shown that fiber orientation played a decisive role in the mechanical properties of composites.

### 3.2. Effect of Injection Pressure on Mechanical Properties

**Figure 14:**
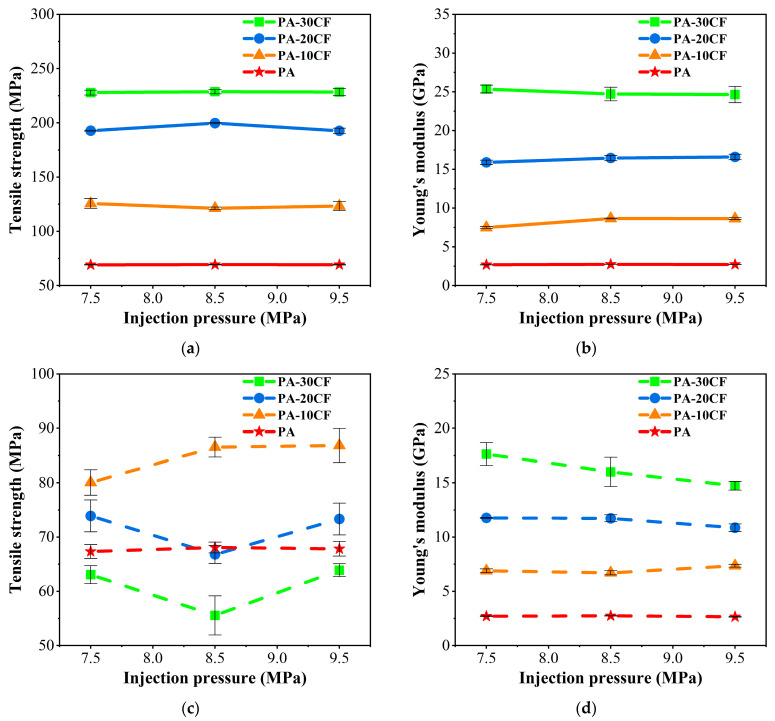
Tensile properties of PA-CF composites for different injection pressures: (**a**) tensile strength, without weld lines; (**b**) Young’s modulus, without weld lines; (**c**) tensile strength, with weld lines; and (**d**) Young’s modulus, with weld lines.

The tensile properties of PA-CF composites were also measured for different injection pressures. As shown in Figure 14a,b, fiber content remained the most significant influencing factor for PA-CF composites without weld lines. When carbon fiber reached 30wt%, the tensile strength and Young’s modulus of PA-CF composites were 2.29 and 8.43 times higher than that of pure nylon, respectively (injection molding at 7.5 MPa). In general, the injection pressure had little effect on the tensile strength and modulus of PA-CF composites without weld lines, which was similar to the results for the injection temperature. The majority of carbon fibers were oriented along the flow direction in the specimens without weld lines, and the influence of injection pressure on fiber orientation was limited as well. Therefore, changing the injection pressure did not significantly change the mechanical properties of PA-CF composites.

As shown in Figure 14c, PA-20CF and PA-30CF composites with weld lines had obviously lower tensile strength than PA-10CF, and PA-30CF was lower than pure nylon, which was similar to the effect of injection temperature. Only a small proportion of carbon fibers passed through the weld line region in samples with higher fiber content, and a large proportion of fibers were oriented perpendicular to the flow direction and did not play a load-bearing role. PA-20CF and PA-30CF composites obtained the lowest tensile strength at 8.5 MPa, while the values of PA-10CF increased with the rise of injection pressure. As shown in Figure 14d, Young’s modulus of PA-30CF composites with weld lines decreased significantly with the increase in injection pressure. Compared with the specimen at 7.5 MPa, PA-30CF decreased by 9.2% and 16.5% at 8.5 MPa and 9.5 MPa, respectively. A large proportion of fibers were arranged parallel to the interface of the weld line for those with high fiber contents at high injection pressures, which had greater adverse influences on the properties of composites and led to a decline in modulus.

**Figure 15:**
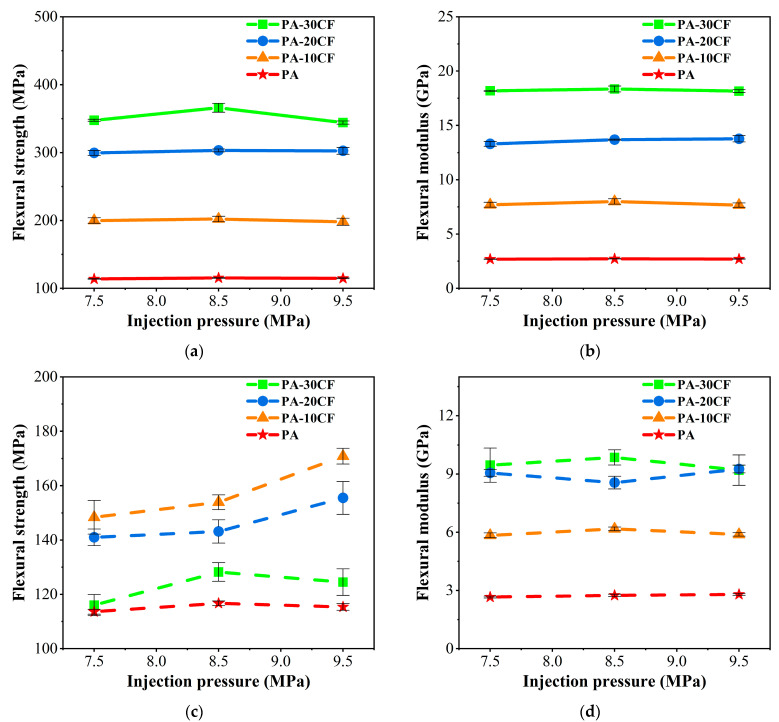
Flexural properties of PA-CF composites for different injection pressures: (**a**) flexural strength, without weld lines; (**b**) flexural modulus, without weld lines; (**c**) flexural strength, with weld lines; and (**d**) flexural modulus, with weld lines.

The flexural properties of PA-CF composites were measured for different injection pressures. As exhibited in Figure 15a,b, the flexural strength and modulus of PA-CF composites without weld lines were increased by 3.05 and 6.76 times, respectively, compared to pure nylon (injection molding at 7.5MPa) when carbon fiber reached 30 wt%. The injection pressure had little effect on the flexural modulus of PA-CF composites without weld lines.

Different from the influences of injection temperature, the flexural strength of PA-CF composites with weld lines increased with the ascent of injection pressure (Figure 15c). PA-10CF composites had the largest flexural strength at each value of injection pressure. Compared with the flexural strength at 7.5 MPa, PA-10CF composites increased by 3.7% and 15.2% at 8.5 MPa and 9.5 MPa, respectively.

Weld line coefficients for different injection pressures were calculated according to Formula (1) $F_{kl}=\frac{\sigma_{WL}}{\sigma_{NWL}}$ and listed in Table 5. The weld line had a more obvious influence on the mechanical properties of PA-CF composites when fiber content increased, which was similar to the results of injection temperature. In addition, the Young’s modulus weld line coefficient of PA-30CF and PA-20CF composites decreased with the ascent of injection pressure. The value of PA-10CF reached 0.92 at 7.5 MPa, indicating Young’s modulus of PA-10CF with weld lines was close to that without weld lines. The flexural strength weld line coefficient of specimens containing fibers increased with the rise of injection pressure.

**Figure 16:**
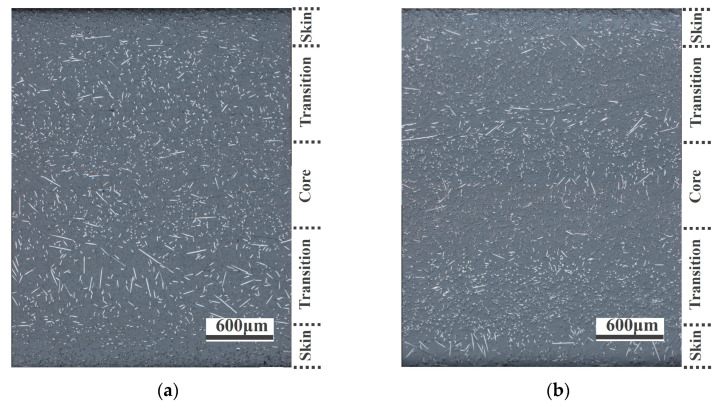
Metallographic micrograph of samples with weld lines: (**a**) PA-10CF, 7.5MPa and (**b**) PA-10CF, 9.5 MPa.

**Figure 17:**
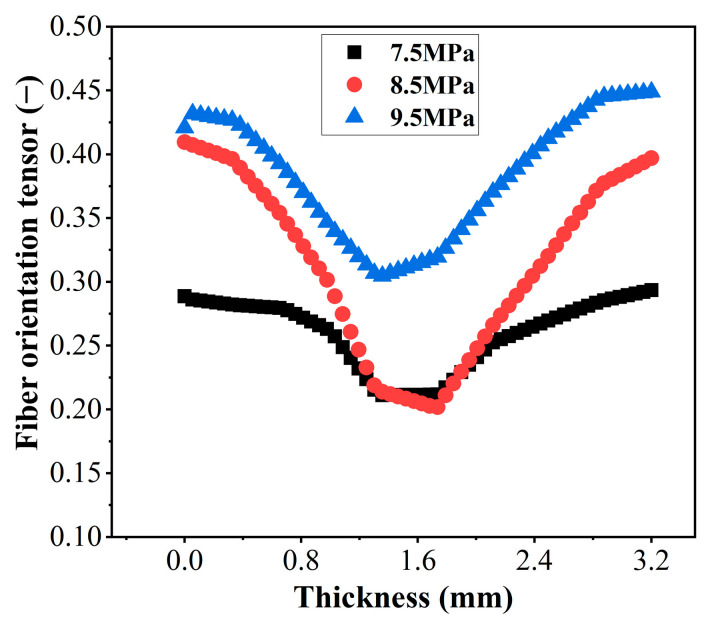
Fiber orientation tensor of PA-10CF with weld lines for different injection pressures.

As shown in Figure 16, to further analyze the influences of injection pressure on microstructure, microscopic images of PA-10CF composites at 7.5 MPa and 9.5 MPa in weld lines regions were observed, showing the structure of the skin–transition–core. It is worth noting that the fibers in the transition layer tend to orient along the flow direction when injection pressure increases, which is beneficial for improving mechanical properties. The transition layer had a significantly better fiber orientation at 9.5 MPa (Figure 16b) than at 7.5 MPa (Figure 16a). The simulation also confirmed this result (Figure 17), indicating that higher injection pressure was conducive to fiber orientation in the weld line region.

## 4. Conclusions

In this work, the effects of injection temperature, injection pressure, and fiber content on the mechanical properties of weld lines were studied for PA-CF composites.

The mechanical properties of pure nylon were hardly influenced by weld lines. The existence of weld lines had negative influences on the mechanical properties of PA-CF composites. The weld line coefficient of PA-CF composites decreased as the fiber content increased, which meant that the damage of weld lines to mechanical properties increased. In weld line regions, there were a large number of fibers distributed vertically to flow direction, which could not play a reinforcing role. Thus, the damaged fractures were also concentrated in these regions. PA-10CF composites with weld lines had the largest mechanical properties due to the proportion of fibers with no reinforcement effect rising as fiber content increased. In addition, increasing the injection temperature and pressure facilitated fiber orientation, which improved the mechanical properties of composites with low fiber content. However, the mechanical properties decreased for composites with high fiber content instead.

## Figures and Tables

**Table 1:** Nominal material properties of SABIC RE0069S.

Properties	Test Standards	Value	Unit
Density	ASTM D792	1.50	g/cm^3^
Tensile strength	ASTM D638	228	MPa
Young’s modulus	ASTM D638	26,960	MPa
Flexural strength	ASTM D790	351	MPa
Flexural modulus	ASTM D790	18,590	MPa

**Table 2:** Injection molding parameters.

Injection Parameters	Value	Unit
Injection temperature	295, 305, 315	°C
Injection pressure	7.5, 8.5, 9.5	MPa
Mold temperature	25	°C
Packing pressure	8	MPa
Injection velocity	70	mm/s

**Table 3:** The values of material parameters.

Parameters	PA-10CF	PA-20CF	PA-30CF
n (−)	0.449	0.474	0.536
$\tau^{*}$ (Pa)	4.83 × 10^4^	1.56 × 10^4^	1.35 × 10^4^
$D_{1}$ (Pa·S)	8.58 × 10^16^	1.68 × 10^18^	1.18 × 10^21^
$D_{2}$ (K)	333.15	343.15	373.15
$D_{3}$ (K/Pa)	0	0	0
$A_{1}$ (−)	42.98	48.13	52.52
$\tilde{A_{2}}$ (K)	51.6	51.6	51.6

**Table 4:** Weld line coefficients for different fiber contents and injection temperatures (Mean ± SD).

Materials	Injection Temperature (°C)	*F_kl_* of Tensile Strength (−)	*F_kl_* of Young’s Modulus (−)	*F_kl_* of Flexural Strength (−)	*F_kl_* of Flexural Modulus (−)
PA	295	0.95 ± 0.04	0.99 ± 0.02	1.00 ± 0.02	1.01 ± 0.02
	305	0.98 ± 0.03	1.00 ± 0.01	1.01 ± 0.02	1.01 ± 0.02
	315	0.99 ± 0.03	1.00 ± 0.02	1.00 ± 0.02	1.01 ± 0.02
PA-10CF	295	0.65 ± 0.01	0.82 ± 0.03	0.60 ± 0.02	0.72 ± 0.03
	305	0.71 ± 0.01	0.77 ± 0.02	0.76 ± 0.03	0.77 ± 0.04
	315	0.67 ± 0.02	0.88 ± 0.02	0.69 ± 0.04	0.76 ± 0.04
PA-20CF	295	0.38 ± 0.02	0.73 ± 0.01	0.53 ± 0.01	0.73 ± 0.01
	305	0.33 ± 0.01	0.71 ± 0.01	0.47 ± 0.02	0.63 ± 0.02
	315	0.32 ± 0.02	0.68 ± 0.01	0.45 ± 0.03	0.61 ± 0.02
PA-30CF	295	0.27 ± 0.03	0.66 ± 0.02	0.34 ± 0.01	0.43 ± 0.01
	305	0.24 ± 0.02	0.65 ± 0.06	0.35 ± 0.01	0.54 ± 0.01
	315	0.25 ± 0.03	0.62 ± 0.09	0.31 ± 0.01	0.50 ± 0.02

**Table 5:** Weld line coefficients for different injection pressures (Mean ± SD).

Materials	Injection Pressure (MPa)	*F_kl_* of Tensile Strength (−)	*F_kl_* of Young’s Modulus (−)	*F_kl_* of Flexural Strength (−)	*F_kl_* of Flexural Modulus (−)
PA	7.5	0.97 ± 0.05	1.01 ± 0.01	1.00 ± 0.01	0.99 ± 0.02
	8.5	0.98 ± 0.03	1.00 ± 0.01	1.01 ± 0.02	1.01 ± 0.02
	9.5	0.98 ± 0.03	0.98 ± 0.02	1.00 ± 0.01	1.04 ± 0.02
PA-10CF	7.5	0.64 ± 0.03	0.92 ± 0.03	0.74 ± 0.05	0.76 ± 0.02
	8.5	0.71 ± 0.01	0.77 ± 0.02	0.76 ± 0.03	0.77 ± 0.04
	9.5	0.70 ± 0.02	0.85 ± 0.02	0.86 ± 0.03	0.77 ± 0.03
PA-20CF	7.5	0.38 ± 0.03	0.74 ± 0.02	0.47 ± 0.01	0.68 ± 0.01
	8.5	0.33 ± 0.01	0.71 ± 0.01	0.47 ± 0.02	0.63 ± 0.02
	9.5	0.38 ± 0.03	0.65 ± 0.02	0.51 ± 0.01	0.67 ± 0.01
PA-30CF	7.5	0.28 ± 0.02	0.70 ± 0.06	0.33 ± 0.02	0.52 ± 0.03
	8.5	0.24 ± 0.02	0.65 ± 0.06	0.35 ± 0.01	0.54 ± 0.01
	9.5	0.28 ± 0.01	0.60 ± 0.07	0.36 ± 0.02	0.51 ± 0.04

## Data Availability

Data are contained within the article.

## References

[1] Fisa B., Dufour J., Vu-Khanh T. (1987). Weld line integrity of reinforced plastics: Effect of filler shape. Polym. Compos..

[2] Kovács J.G., Sikló B. (2010). Experimental validation of simulated weld line formation in injection moulded parts. Polym. Test..

[3] Hashemi S. (2011). Strength of single- and double-gated injection moulded short glass fibre reinforced polycarbonate. J. Thermoplast. Compos. Mater..

[4] Khamsehnezhad A., Hashemi S. (2008). Mechanical properties of single- and double-gated injection moulded short glass fibre reinforced PBT/PC composites. J. Mater. Sci..

[5] Li H., Guo Z., Li D. (2007). Reducing the effects of weld lines on appearance of plastic products by Taguchi experimental method. Int. J. Adv. Manuf. Technol..

[6] Gim J., Turng L.-S. (2022). A review of current advancements in high surface quality injection molding: Measurement, influencing factors, prediction, and control. Polym. Test..

[7] Baradi M.B., Cruz C., Riedel T., Régnier G. (2019). Frontal weld lines in injection-molded short fiber-reinforced PBT: Extensive microstructure characterization for mechanical performance evaluation. Polym. Compos..

[8] Baradi M.B., Cruz C., Riedel T., Régnier G. (2019). Mechanical and microstructural characterization of flowing weld lines in injection-molded short fiber-reinforced PBT. Polym. Test..

[9] Lim J.K., Shoji T. (1993). Fiber orientation of polymer injection weld and its strength evaluation. KSME J..

[10] Chen C.-S., Chen T.-J., Chien R.-D., Chen S.-C. (2007). Investigation on the weld line strength of thin-wall injection molded ABS parts. Int. Commun. Heat Mass Transf..

[11] Daiyan H., Andreassen E., Grytten F., Lyngstad O.V., Luksepp T., Osnes H. (2010). Low-velocity impact response of injection-moulded polypropylene plates—Part 2: Effects of moulding conditions, striker geometry, clamping, surface texture, weld line and paint. Polym. Test..

[12] Dzulkipli A.A., Azuddin M. (2017). Study of the Effects of Injection Molding Parameter on Weld Line Formation. Procedia Eng..

[13] Kagitci Y.C., Tarakcioglu N. (2016). The effect of weld line on tensile strength in a polymer composite part. Int. J. Adv. Manuf. Technol..

[14] Malguarnera S.C., Manisali A. (1981). The effects of processing parameters on the tensile properties of weld lines in injection molded thermoplastics. Polym. Eng. Sci..

[15] Chrysostomou A., Hashemi S. (1998). Mechanical properties of injection moulded styrene maleic anhydride (SMA) Part II Influence of short glass fibres and weld lines. J. Mater. Sci..

[16] Fisa B., Rahmani M. (1991). Weld line strength in injection molded glass fiber-reinforced polypropylene. Polym. Eng. Sci..

[17] Hashemi S. (2007). Thermal effects on weld and unweld tensile properties of injection moulded short glass fibre reinforced ABS composites. Express Polym. Lett..

[18] Scantamburlo A., Zanini F., Lucchetta G., Sorgato M. (2022). Improving the weld lines mechanical properties by combining alternate dynamic packing and rapid heat cycle moulding. Compos. Part A Appl. Sci. Manuf..

[19] Ozcelik B., Kuram E., Topal M.M. (2012). Investigation the effects of obstacle geometries and injection molding parameters on weld line strength using experimental and finite element methods in plastic injection molding. Int. Commun. Heat Mass Transf..

[20] Oh G.-H., Jeong J.-H., Park S.-H., Kim H.-S. (2018). Terahertz time-domain spectroscopy of weld line defects formed during an injection moulding process. Compos. Sci. Technol..

[21] Martulli L.M., Kerschbaum M., Lomov S.V., Swolfs Y. (2020). Weld lines in tow-based sheet moulding compounds tensile properties: Morphological detrimental factors. Compos. Part A Appl. Sci. Manuf..

[22] Seldén R. (1997). Effect of processing on weld line strength in five thermoplastics. Polym. Eng. Sci..

[23] Choudhari D.S., Kakhandki V.J. (2021). Comprehensive study and analysis of mechanical properties of chopped carbon fibre reinforced nylon 66 composite materials. Mater. Today Proc..

[24] Guo A., Liu C., Li S., Zhou X., Wang J., Wang S., Qu P., Hu Y. (2022). Water absorption rates and mechanical properties of material extrusion-printed continuous carbon fiber-reinforced nylon composites. J. Mater. Res. Technol..

[25] Ma Y., Jin S., Yokozeki T., Ueda M., Yang Y., Elbadry E.A., Hamada H., Sugahara T. (2020). Effect of hot water on the mechanical performance of unidirectional carbon fiber-reinforced nylon 6 composites. Compos. Sci. Technol..

[26] Quintana M.C., Frontini P. (2020). Weld line strength factors in a reinforced injection molded part: Relationship with predicted fiber orientation. J. Reinf. Plast. Compos..

[27] Cao W., Shen Y., Wang P., Yang H., Zhao S., Shen C. (2019). Viscoelastic modeling and simulation for polymer melt flow in injection/compression molding. J. Non-Newton. Fluid Mech..

[28] Trotta G., Stampone B., Fassi I., Tricarico L. (2021). Study of rheological behaviour of polymer melt in micro injection moulding with a miniaturized parallel plate rheometer. Polym. Test..

[29] Xu X., Tian L., Peng S., Yu P. (2022). Development of SPH for simulation of non-isothermal viscoelastic free surface flows with application to injection molding. Appl. Math. Model..

[30] Thakre P., Chauhan A.S., Satyanarayana A., Raj Kumar E., Pradyumna R. (2018). Estimation of Shrinkage & Distortion in Wax Injection using Moldex3D Simulation. Mater. Today Proc..

[31] Gao P. (2022). Three dimensional finite element computation of the non-isothermal polymer filling process by the phase field model. Adv. Eng. Softw..

[32] Kuo H.-C., Jeng M.-C. (2010). Effects of part geometry and injection molding conditions on the tensile properties of ultra-high molecular weight polyethylene polymer. Mater. Des..

[33] Hashemi S., Lepessova Y. (2007). Temperature and weldline effects on tensile properties of injection moulded short glass fibre PC/ABS polymer composite. J. Mater. Sci..

[34] Vaxman A., Narkis M., Siegmann A., Kenig S. (1991). Weld-line characteristics in short fiber reinforced thermoplastics. Polym. Compos..

[35] Solymossy B., Kovacs J. (2008). The Examination of Weld Line Properties in Injection Molded PP Composites. Mater. Sci. Forum.

[36] Wu W., Zhao B., Mo F., Li B., Jiang B. (2022). In-line steady shear flow characteristics of polymer melt in rectangular slit cavities during thin-wall/micro injection molding. Mater. Des..

[37] Wittemann F., Kärger L., Henning F. (2021). Theoretical approximation of hydrodynamic and fiber-fiber interaction forces for macroscopic simulations of polymer flow process with fiber orientation tensors. Compos. Part C Open Access.

[38] Sasayama T., Sato N., Katagiri Y., Murayama Y. (2020). Particle-level simulation for the prediction of short fiber orientation in injection molding. Compos. Part A Appl. Sci. Manuf..

